# A novel *RAB39B* mutation and concurrent de novo *NF1* mutation in a boy with neurofibromatosis type 1, intellectual disability, and autism: a case report

**DOI:** 10.1186/s12883-020-01911-0

**Published:** 2020-09-01

**Authors:** Claudia Santoro, Teresa Giugliano, Pia Bernardo, Federica Palladino, Annalaura Torella, Francesca del Vecchio Blanco, Maria Elena Onore, Marco Carotenuto, Vincenzo Nigro, Giulio Piluso

**Affiliations:** 1 Department of Physical and Mental Health, and Preventive Medicine, University of Campania “Luigi Vanvitelli”, Naples, Italy; 2 Department of Women, Children, and General and Specialized Surgery, University of Campania “Luigi Vanvitelli”, Naples, Italy; 3 Department of Precision Medicine, University of Campania “Luigi Vanvitelli”, Via Luigi De Crecchio,7 –, 80138 Naples, Italy; 4 Department of Neurosciences, Pediatric Hospital Santobono-Pausilipon, Naples, Italy; 5 Telethon Institute of Genetics and Medicine (TIGEM), Pozzuoli, Italy

**Keywords:** Neurofibromatosis type 1, *RAB39B*, X-linked intellectual disability, Autism, Parkinson’s disease, Case report

## Abstract

**Background:**

Mutations in *RAB39B* at Xq28 causes a rare form of X-linked intellectual disability (ID) and Parkinson’s disease. Neurofibromatosis type 1 (NF1) is caused by heterozygous mutations in *NF1* occurring de novo in about 50% of cases, usually due to paternal gonadal mutations. This case report describes clinical and genetic findings in a boy with the occurrence of two distinct causative mutations in *NF1* and *RAB39B* explaining the observed phenotype.

**Case presentation:**

Here we report a 7-year-old boy with multiple café-au-lait macules (CALMs) and freckling, severe macrocephaly, peculiar facial gestalt, severe ID with absent speech, epilepsy, autistic traits, self-harming, and aggressiveness. Proband is an only child born to a father aged 47. Parents did not present signs of NF1, while a maternal uncle showed severe ID, epilepsy, and tremors.By RNA analysis of *NF1*, we identified a de novo splicing variant (NM_000267.3:c.6579+2T>C) in proband, which explained NF1 clinical features but not the severe ID, behavioral problems, and aggressiveness. Family history suggested an X-linked condition and massively parallel sequencing of X-exome identified a novel *RAB39B* mutation (NM_171998.2:c.436_447del) in proband, his mother, and affected maternal uncle, subsequently validated by Sanger sequencing in these and other family members.

**Conclusions:**

The case presented here highlights how concurrent genetic defects should be considered in NF1 patients when *NF1* mutations cannot reasonably explain all the observed clinical features.

## Background

Mutations in *RAB39B* (MIM 300774) at Xq28 cause a syndromic form of X-linked intellectual disability (XLID), with very few affected males described to date (MRX72; MIM 300271) [1–3].

Affected patients present variable neurological features, including moderate to severe ID, seizures, autism spectrum disorder (ASD), macrocephaly, delayed psychomotor development, and early-onset Parkinson’s disease, as in the allelic Waisman syndrome (WSMN; OMIM 311510) [2].

In mouse brain, *Rab39b* is expressed in cortical and hippocampal neurons, as well as in dopaminergic neurons of the substantia nigra, concordant with its association with parkinsonism and cognitive impairment in humans [4]. *Rab39b* knockout mice showed reduced cortical neurogenesis, macrocephaly, and autistic behaviors, similarly to patients with mutations in *RAB39B* [5]. The Ras-related protein Rab-39B is a small neuron-specific GTPase that contributes to synapse formation and maintenance by regulating organization and dynamics of intracellular membranes and vesicular membrane traffic [6]. With its effector, the protein interacting with C-kinase 1, Rab-39B regulates availability of AMPA receptor, important for synaptic plasticity [7]. Furthermore, the complex formed by C9orf72, WDR41, and SMCR8 was found to act as a GDP/GTP exchange factor for Rab-8A and Rab-39B, suggesting that Rab-39B might be involved in autophagy regulation [8]. However, precisely how *RAB39B* loss-of-function or increased dosage [9] can perturb neuronal development leading to cognitive impairment needs further clarification.

Neurofibromatosis type 1 (NF1; MIM 162200) is caused by dominantly inherited mutations in *NF1* (MIM 613113), a complex gene encoding for neurofibromin, a GTPase-activating protein that negatively regulates Ras/MAPK signaling pathway [10]. In about 50% of cases, mutations occur de novo, with *NF1* exhibiting one of the highest single locus mutation rates known in humans (1 × 10^− 4^ per gamete per generation) [11]. De novo mutations are mainly linked to paternal age and to the increasing number of cell divisions in the male germ line [12].

Here, we describe a boy with clinical diagnosis of NF1 complicated by severe macrocephaly, dysmorphic features, severe ID with absent speech, epilepsy, and ASD. Based on his family history, these neurological features could not be only assigned to NF1 and an extensive genetic characterization was carried out. To our knowledge, this is the first case in which two distinct causative mutations in *NF1* and *RAB39B* explained the observed phenotype.

## Case presentation

**Fig. 1:**
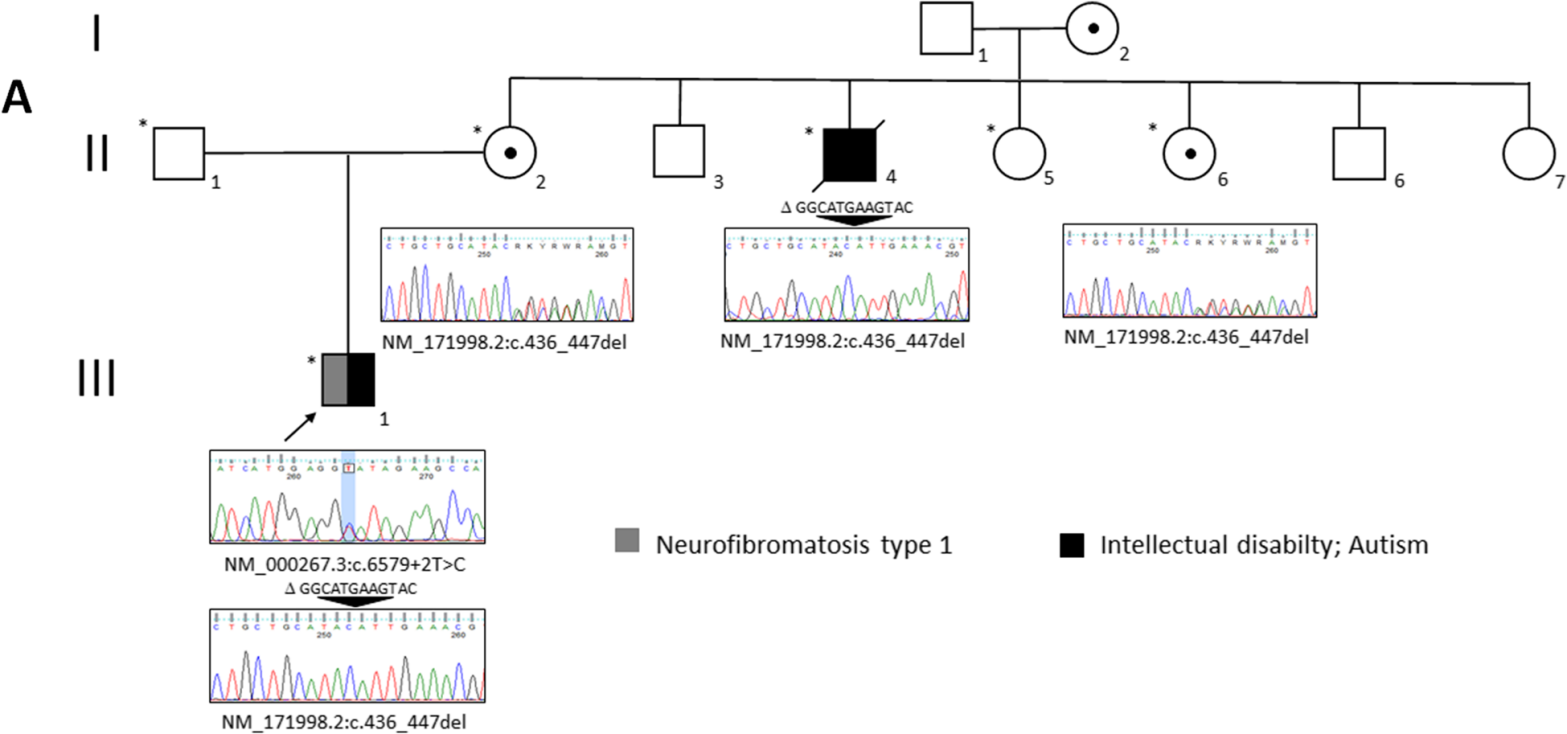
*Pedigree and clinical features of proband with concurrent RAB39B and NF1 mutations*. An asterisk designates subjects recruited for the genetic study; an arrow indicates proband. Sequence data for mutant alleles and their family segregation are shown below the symbol

We report the clinical and genetic characterization of a boy (III.1; Fig. 1), the only child of apparently healthy parents, who was referred to our NF1 Referral Center at age 7 due to the presence of CALMs and freckling.

Proband was born at week 39 of an uneventful pregnancy by C-section because of neonatal macrosomia. Prenatal karyotyping was normal (46, XY). At birth, weight was 3.930 kg (75th centile), length 52 cm (75th centile), and head circumference 35 cm (66th centile). The child presented respiratory distress not requiring intubation. He started walking at 2 years and spoke his first words at 12 months, with reported regression and loss of verbal competence soon after.

Severe ID and ASD was diagnosed at age 3, and pharmacologically treated with risperidone. At this age, he had his first episode of generalized seizures during fever, followed by others while awake and in apyrexia. A sleep electroencephalogram showed rare physiological figures, as sleep spindles and k complex, and epileptiform abnormalities in the right temporal region. Seizures were adequately controlled by levetiracetam. Brain magnetic resonance imaging performed at age 4 showed periventricular white matter hyperintensities, likely due to hypoxia, and revealed T2W hyperintensities in basal ganglia bilaterally, while FLAIR images showed hippocampal and temporomesial hyperintensities bilaterally. Ophthalmological evaluation, electrocardiogram, auditory/visual brainstem potentials were normal. He showed hyperactivity and motor instability, as well as limited social interactions and aggressiveness. At age 6, sleep disorder (insomnia) was observed and chlorpromazine, clonazepam, and melatonin were added to the pharmacological therapy.

At our first consultation, family history revealed that a maternal uncle (II.4; Fig. 1) was also affected by severe ID, absence of speech, and tremors, with global motor impairment. He had been institutionalized for many years and while this study was ongoing died suddenly at the age of 53 from complications of a pulmonary infection, preventing any further clinical examination. Proband was still able to walk, and epilepsy and sleep disorders were well managed pharmacologically. Global motor impairment, severe behavioral disorders, including self-injurious behavior and psychomotor agitation episodes, and food selectivity were noted. At medical examination, he presented with CALMs and freckling, suggestive of NF1 according to NIH diagnostic criteria [10]. He presented restless with absent speech (less than 5 words), macrocephaly (head circumference 56 cm, > +2SD), peculiar facial gestalt, gingival hypertrophy, and generalized hypotonia. Previously performed array-CGH and genetic testing for fragile X syndrome resulted negative.

At last observation, at age 14, his pharmacological therapy included the oral administration of risperidone (0.75 mg BID), levetiracetam (250 mg BID), lorazepam (6 drops BID), clonazepam (12 drops BID) and sodium valproate (250 mg TID), with a moderate control of both seizures and behavioral problems.

Based on clinical signs of NF1 and family history suggestive of an XLID, despite suspicion of neonatal hypoxia, a comprehensive genetic investigation was performed. As the legal representative of a minor, proband’s mother gave written informed consent to the study.

RNA analysis (see “Supplemental Information”) [13] revealed skipping of exon 43 in *NF1* transcript (not shown) due to a de novo heterozygous splicing variant at genomic level [NM_000267.3:c.6579+2T>C; p.(Glu2122Glyfs*27)] (Fig. 1). X-exome sequencing was then performed on proband, his mother, and affected maternal uncle (see “Supplemental Information”) [14]. We identified a novel *RAB39B* mutation (NM_171998.3:c.436_447del) (Fig. 1). This *in-frame* deletion removed four amino acids (p.Gly146_Tyr149del) in the C-terminus region of Ras-related protein Rab-39B. Segregation analysis in family members was in line with X-linked inheritance and one (II.6) of two maternal aunts participating in the study resulted a carrier (Fig. 1). As unbalanced X inactivation is reported in females, [2, 3, 15] proband’s mother and maternal aunts were further investigated, highlighting preferential inactivation of the mutant allele in carrier females (II.2 66%:34% and II.6 73%:27%, respectively).

## Discussion and conclusions

Cognitive impairment commonly affects children with NF1 and includes an IQ in the low average range, learning difficulties and social dysfunction [16, 17]. ID was reported for NF1 patients carrying a 17q11.2 microdeletion, [18] while ASD features were observed in up to 30% of children with NF1 and epilepsy in about 4% of NF1 patients [19, 20].

We identified a de novo heterozygous mutation in *NF1* that affects splicing and was not related to a specific NF1 phenotype or to an impaired cognitive profile [13]. Severe ID with absent speech, associated with epilepsy, ASD, self-injurious behavior, and aggressiveness, could not be explained by the *NF1* mutation alone. Although brain imaging showed signs of hypoxia, family history suggested the existence of an additional genetic condition, likely linked to X-chromosome.

**Fig. 2:**
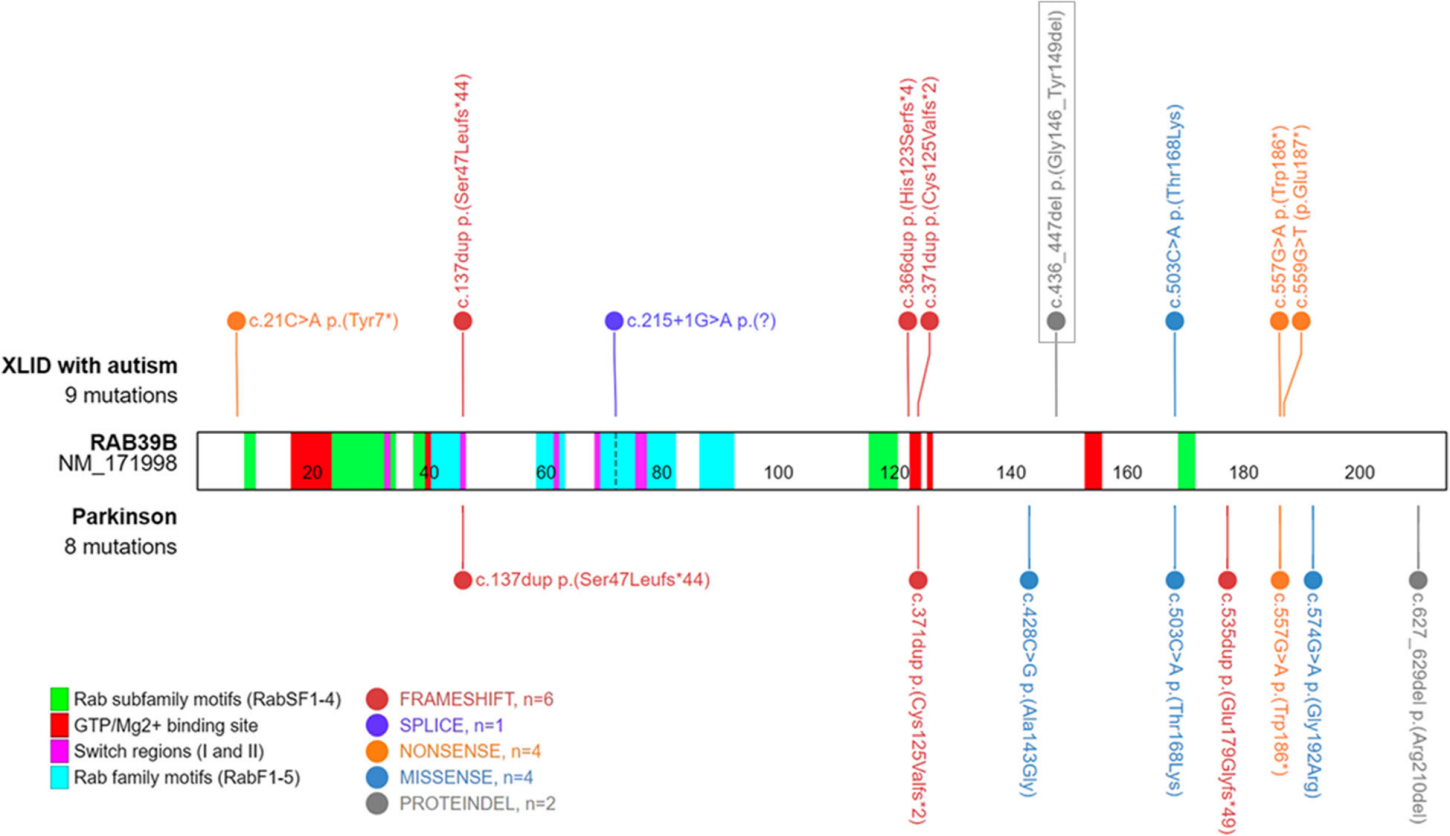
*Graphic view of Rab-39B with its functional domains and published pathogenic variants.* Functional motifs of Rab-39B (213 amino acids) are differently colored. Pathogenic variants associated with ID and autism (top) or parkinsonism (bottom) are color grouped according to their functional effect. The variant presented here is boxed

A novel *RAB39B* mutation (NM_171998.3:c.436_447del) was subsequently identified in proband, his carrier mother, and affected maternal uncle, as well as in one maternal aunt (Fig. 1). This *in-frame* deletion removed four amino acids (p.Gly146_Tyr149del) in the C-terminus region of Rab-39B. To date, only 12 causative variants were reported in *RAB39B*, mainly associated with variable ID, macrocephaly, and ASD, and in some cases complicated by early-onset Parkinson’s disease (Fig. 2) [1–3, 15, 21–23].

**Fig. 3:**
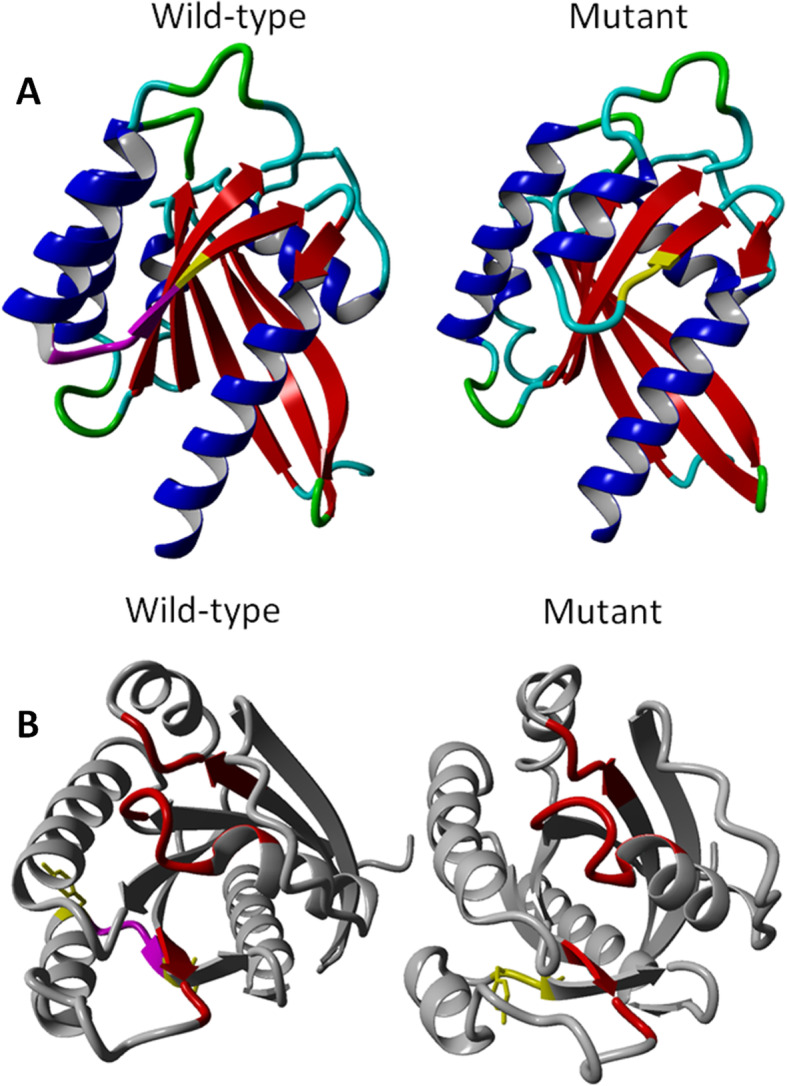
*3D homology modeling*. The 3D homology model (RefSeq: NP_741995.1; residues 1–213) was generated based on the available Rab-8A model (RCS-PDB: 5SZI; residues 1–209) for wild-type and mutant form. **a** Deleted residues (GMKY) are highlighted in magenta, and flanking residues (Y^145^; I^150^) in yellow. **b** The GTP/Mg2+ binding site is highlighted in red

As other Ras-related GTPases, Rab proteins have six β-sheets and two Switch elements (I and II) that change conformation upon nucleotide binding (Fig. 2) [24]. They also present five specific Rab family (F1–5) and four Rab subfamily (SF1–4) motifs (Fig. 2) [24]. Three-dimensional homology modeling [25] based on the available Ras-related protein Rab-8A model (RCS-PDB: 5SZI) showed that the mutant protein lost part of the sixth β-sheet and the nearby α-helix compared to wild-type (Fig. 3a). GTP/Mg^2+^ binding site conformation also seemed to be affected (Fig. 3b) [26].

The mutation identified in *RAB39B* explained neurocognitive and neurological features observed in affected male family members, while the unbalanced X inactivation in carrier females was in agreement with their apparently healthy status. The independent occurrence of a de novo *NF1* mutation accounted for NF1 signs and could contribute to the severity of neurocognitive features.

In NF1, severe cognitive impairment not linked to a 17q11.2 microdeletion syndrome or not explainable by other causes should not exclude the albeit rare possibility of another concomitant genetic condition. The high mutation rate of *NF1* and the relatively not low worldwide prevalence of neurofibromatosis type 1, can explain the occurrence with NF1 of distinct genetic condition in the same patient [27–30]. This case report clearly underscores the need for more extensive genetic investigation, today possible thanks to massive clinical application of next-generation sequencing-based genetic testing, when the identification of a causative mutation does not fully explain the observed phenotype.

## Supplementary information

**Additional file 1.**

## Data Availability

All data generated or analyzed during this study are included in this published article.

## Abbreviations

AMPA: α-amino-3-hydroxy-5-methyl-4-isoxazolepropionic acid
ASD: Autism spectrum disorder
BID: Twice a day
CALMs: Café-au-lait macules
FLAIR: Fluid attenuation inversion recovery
ID: Intellectual disability
NF1: Neurofibromatosis type 1
RAB39B: Gene encoding Ras-related protein Rab-39B
Ras/MAPK: Ras/mitogen activated protein kinase
TID: Three times a day
XLID: X-linked intellectual disability
