# Supplementary material for: A novel RAB39B mutation and concurrent de novo NF1 mutation in a boy with neurofibromatosis type 1, intellectual disability, and autism: a case report
Source: BMC Neurol. 2020 Sep 1;20:327. doi: 10.1186/s12883-020-01911-0 (PMC7460788; doi:10.1186/s12883-020-01911-0)
Supplement: Supplementary file 1 — Additional file 1. [file 12883_2020_1911_MOESM1_ESM.docx]

**Materials and Methods**

*NF1 mutation screening*

Briefly, total RNA and genomic DNA was extracted from leukocytes of peripheral blood samples using standard procedures. The entire coding sequences of *NF1* (RefSeq: NM_000267) was then amplified in twenty partially overlapping fragments of about 500–700 bp, which were bidirectionally sequenced on an ABI 3130xL automatic DNA sequencer (Life Technologies, Carlsbad, California, USA).

*X-exome sequencing*

For the proband, his mother and affected maternal uncle, enrichment of the target sequences of almost all the coding genes on X-chromosome was performed using the Agilent SureSelectXT X-Chromosome in-solution target enrichment kit (Agilent Technologies, Santa Clara, California, USA) according to the manufacturer’s instructions.

Sequencing was performed using a NextSeq 500 system (Illumina, San Diego, California, USA). On average, targeted resequencing generated 19.7 Mb of sequence data as 150 bp paired-end reads. The mean coverage of targeted regions was 99.5% at 10x and 98.3% at 50x, ensuring the detection of genetic variants with high sensitivity and specificity. Sequence data were analyzed using an in-house pipeline designed to automate the analysis workflow. Paired sequencing reads were aligned to the reference genome (UCSC, hg19 build) using Burrows-Wheeler Aligner, and sorted with SAMtools and Picard (http://picard.sourceforge.net). Calling of single nucleotide variants (SNVs) and small insertions/deletions (Ins/Del) was performed with Genome Analysis Toolkit (GATK). The called SNVs and Ins/Del variants were annotated using ANNOVAR.

For data filtering, we considered: (1) variants that passed quality control and with more than 10 reads; (2) variants with a frequency < 1% in the global and European population, as well as in Genome Aggregation Database (<https://gnomad.broadinstitute.org/>) and in ExAC Browser (<http://exac.broadinstitute.org/>); (3) variants that were not annotated in dbSNP; (4) hemizygous variants in the proband and affected maternal uncle, segregating in agreement with an X-linked inheritance; (5) variants with a potential effect on gene function.

*Variant validation*

Exon 43 of *NF1* and exon 2 of *RAB39B*, with their flanking regions, were amplified by PCR on genomic DNA from proband and his relatives. PCR products were double-strand sequenced using BigDye Terminator sequencing chemistry (Life Technologies, Carlsbad, California, USA) and analyzed on an ABI 3130xL automatic DNA sequencer (Life Technologies, Carlsbad, California, USA).

*3D homology modeling*

Three-dimensional homology modeling of Rab-39B was based on the available Rab-8B model (RCS-PDB: 5SZI) and generated using SWISS-MODEL (<https://swissmodel.expasy.org/>).
